# Tick Microbiome and Its Role in Emerging Zoonotic Diseases and Transmissibility

**DOI:** 10.3390/microorganisms14061281

**Published:** 2026-06-05

**Authors:** So Youn Youn, Hyang-Sim Lee, Mi-Sun Yoo, Yun Sang Cho

**Affiliations:** Bacterial Disease Division, Department of Animal and Plant Health Research, Animal and Plant Quarantine Agency, 177, Hyeoksin 8-ro, Gimcheon-si 39660, Gyeongsangbuk-do, Republic of Korea; syyoun@korea.kr (S.Y.Y.); leehs76@korea.kr (H.-S.L.); msyoo99@korea.kr (M.-S.Y.)

**Keywords:** tick microbiome, zoonotic diseases, vector-borne diseases, pathogen transmission, emerging infectious diseases, One Health, tick vaccines

## Abstract

Ticks are important arthropod vectors that transmit various pathogens to humans, livestock, and wildlife, thereby contributing significantly to the global burden of vector-borne diseases. The tick microbiome, consisting of bacteria, viruses, protozoa, and other microorganisms, plays a crucial role in pathogen transmission dynamics and the emergence of new zoonotic diseases. This review examines the characteristics of tick vectors, the composition and dynamics of tick-associated microbiomes, and their implications for zoonotic disease transmission. We analyze current knowledge of tick-borne pathogens, including *Borrelia burgdorferi* sensu lato, *Rickettsia* species, *Anaplasma* species, and *Coxiella* species, and highlight the potential for microbiome constituents to serve as reservoirs for emerging pathogens. The complex interactions between tick hosts, their microbiomes, and vertebrate hosts create opportunities for pathogen evolution and interspecies transmission. Recent advances in molecular techniques have revealed previously unknown microbial diversity within tick populations, suggesting that many potential zoonotic pathogens remain undiscovered. We discuss future research directions, including field screening methodologies for pathogen detection, microbiome-based risk assessment approaches, and the development of novel prevention strategies, including tick vaccines.

## 1. Introduction

**Figure 1:**
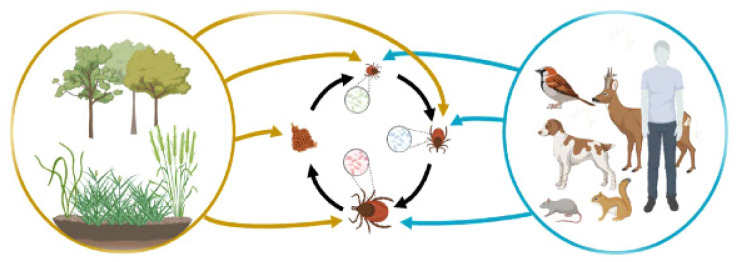
Tick microbiome and pathogen transmission mechanisms. Diagram illustrating the composition of the tick microbiome and its effects on pathogen transmission. Shows interactions between microbiome components and the processes of pathogen acquisition, maintenance, and transmission.

Ticks are among the most important arthropod vectors globally from medical and veterinary perspectives, ranking second only to mosquitoes in their capacity to transmit pathogens to humans and animals [1,2]. These obligate blood-feeding ectoparasites vector a remarkable diversity of microorganisms, including bacteria, viruses, protozoa, and helminths, many of which cause significant morbidity and mortality in vertebrate hosts (Figure 1) [3,4]. The global burden of tick-borne diseases continues to increase due to factors including climate change, land-use changes, wildlife population dynamics, and expanding human activities in natural habitats [5].

The concept of the tick microbiome has emerged as a critical factor in understanding pathogen transmission dynamics and the potential for new zoonotic disease emergence [6]. Unlike simple mechanical vectors, ticks maintain complex microbial communities that can influence the acquisition, development, and transmission of pathogens [7]. These microbial communities include not only known pathogenic organisms but also diverse symbionts, commensals, and potentially pathogenic microorganisms whose roles in disease transmission are poorly understood.

## 2. Characteristics of Tick Vectors

### 2.1. Global Occurrence of Tick-Borne Diseases

#### 2.1.1. Livestock

Tick-borne diseases in livestock represent a significant economic burden globally, with annual losses estimated in the billions of dollars. The economic impact extends beyond direct mortality to include reduced milk production, weight loss, reproductive failures, and treatment costs. *Rhipicephalus* species are primary vectors of bovine babesiosis and anaplasmosis, causing significant mortality and reduced productivity in the cattle population. Bovine babesiosis, caused by *Babesia bovis* and *B. bigemina*, results in acute hemolytic anemia, fever, and, in naïve cattle populations, often death [8]. The disease is particularly severe in European breeds introduced to tropical areas where they lack natural immunity [9].

Anaplasmosis, caused by *Anaplasma marginale*, affects red blood cells and causes severe anemia, particularly in adult cattle [10,11]. The disease can persist as chronic infections, making affected animals lifelong carriers. In tropical and subtropical regions, East Coast fever, caused by *Theileria parva* and transmitted by *Rhipicephalus appendiculatus*, remains one of the most devastating tick-borne diseases affecting cattle [12]. The disease causes lymphoproliferative syndrome with mortality rates exceeding 90% in susceptible cattle breeds.

Heartwater disease, caused by *Ehrlichia ruminatium* and transmitted by *Amblyomma* species, affects ruminants across sub-Saharan Africa and some Caribbean islands [13]. The disease is characterized by hydropericardium, pulmonary edema, and neurological signs, with case fatality rates up to 60% in naïve animals.

#### 2.1.2. Companion Animals

Companion animals face increasing risks from tick-borne pathogens, with canine ehrlichiosis, babesiosis, and Lyme disease being major concerns for pet owners and veterinarians [14]. Canine ehrlichiosis manifests in three phases: acute, subclinical, and chronic [15]. The acute phase involves fever, lethargy, and lymphadenopathy, while chronic cases may develop severe thrombocytopenia, anemia, and bleeding disorders. The brown dog tick, *Rhipicephalus sanguineus*, serves as a vector for multiple pathogens affecting dogs, including *Ehrlichia canis* and *Babesia canis* [16]. This tick species is particularly problematic because it can complete its entire life cycle indoors, enabling year-round transmission.

Canine babesiosis presents as acute hemolytic anemia with symptoms including weakness, pale mucous membranes, and dark-colored urine. *Babesia canis* typically causes more severe disease than *B. gibsoni*, with higher mortality rates in untreated cases. The expansion of *Ixodes scapularis* populations has increased the incidence of Lyme disease in dogs throughout North America, with implications for human health due to shared exposure risks [17]. Dogs serve as sentinels for human exposure risk, as they share similar outdoor activities and tick exposure patterns with their owners.

Rocky Mountain spotted fever in dogs is often more severe than in humans, with rapid progression and high case fatality rates if untreated. Clinical signs include fever, neurological abnormalities, and petechial hemorrhages. Feline tick-borne diseases are less common but include tularemia, cytauxzoonosis, and ehrlichiosis, with cats generally showing milder clinical signs than dogs [18].

#### 2.1.3. Wildlife

Wildlife populations serve as important reservoir hosts for tick-borne pathogens, maintaining enzootic transmission cycles that can spill over into livestock and human populations [19]. The reservoir competence of different wildlife species varies significantly, with some species serving as amplifying hosts while others may be dead-end hosts. Small mammals such as white-footed mice (*Peromyscus leucopus*) are highly competent reservoirs for *Borrelia burgdorferi* sensu lato (s.l.), maintaining high spirochete loads that facilitate transmission to feeding ticks [20,21,22].

White-tailed deer populations in North America serve as essential maintenance hosts for adult *Ixodes scapularis* ticks, supporting the transmission cycles of Lyme disease spirochete [23]. While deer are not competent reservoirs for *B. burgdorferi* s.l., they provide essential blood meals for adult ticks and significantly influence tick population dynamics. High deer densities correlate with increased tick abundance, though the relationship is complex and influenced by landscape factors.

Migratory birds play important roles in the long-distance dispersal of infected ticks, contributing to the geographic spread of tick-borne pathogens [24,25]. Passerine birds can transport *Ixodes* ticks across continental distances, introducing pathogens to new geographic areas. Some bird species, particularly ground-feeding species such as thrushes, can harbor high tick burdens during migration. Marine birds have been implicated in the dispersal of *Ixodes uriae* and associated pathogens across oceanic distances.

Wild ungulates, including elk, moose, and roe deer, serve as hosts for various tick species and their pathogens. These large mammals can maintain high tick populations and may serve as mixing vessels for different pathogen strains. Rodent communities serve as reservoirs for numerous tick-borne pathogens, and species composition and density influence pathogen prevalence and diversity within tick populations.

### 2.2. One Health Approach

The One Health concept recognizes that tick-borne diseases exist at the interface of human, animal, and environmental health, requiring integrated surveillance and control strategies [26,27]. This interdisciplinary approach emphasizes collaboration between human medicine, veterinary medicine, and environmental science to address complex health challenges. Successful One Health implementations for tick-borne diseases include coordinated surveillance systems that monitor pathogen prevalence in humans, domestic animals, wildlife, and tick populations simultaneously.

Climate change effects on tick distribution and activity patterns impact disease transmission dynamics in all host species [28,29,30]. Rising temperatures extend tick activity seasons and enable geographic expansion into previously unsuitable habitats [31,32,33]. Changes in precipitation patterns affect tick survival and questing behavior, while extreme weather events can disrupt established transmission cycles [34]. More importantly, large-scale gradients of temperature and atmospheric waste balance play a major role of tick occurrence patterns [35]. Phenological mismatches between ticks and their hosts may alter transmission efficiency and pathogen prevalence.

Landscape changes, including deforestation, urbanization, and agricultural expansion, alter tick–host–pathogen interactions and create new opportunities for zoonotic transmission [36,37]. Forest fragmentation increases edge habitats that favor certain tick species and brings humans into closer contact with infected tick populations. Urban expansion into natural areas creates interfaces where domestic animals, wildlife, and humans share tick exposure risks. Agricultural practices influence host availability and habitat suitability for different tick species.

Effective One Health approaches require shared databases, standardized diagnostic protocols, and coordinated response strategies across sectors [38]. Early warning systems that integrate environmental monitoring, wildlife surveillance, and clinical data can help predict and prevent disease outbreaks. Public health interventions must consider the complex ecological factors that influence tick-borne disease transmission [39].

## 3. Tick-Borne Zoonotic Diseases

### 3.1. Bacterial Pathogens

Bacterial tick-borne zoonoses represent a diverse group of diseases with varying clinical presentations and geographic distributions (Table 1). These pathogens have evolved sophisticated mechanisms to survive in both tick vectors and vertebrate hosts, often requiring complex transmission cycles involving multiple host species.

**Figure 2:**
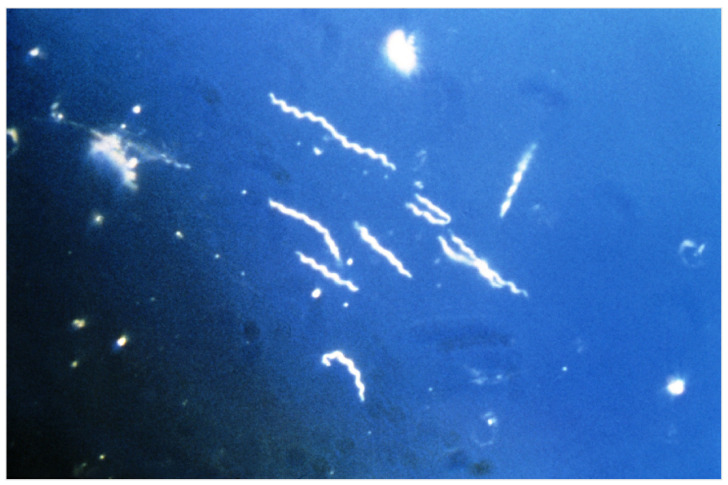
*Borrelia burgdorferi* s.l. spirochete bacteria. Microscopic image of *B. burgdorferi* s.l., the causative agent of Lyme disease. This bacterium, showing a characteristic spiral structure, is one of the most important zoonotic pathogens transmitted by ticks. Source: CDC Public Health Image Library (PHIL) via Wikimedia Commons.

Lyme disease, caused by *B. burgdorferi* s.l., is the most commonly reported tick-borne disease in temperate regions of North America and Europe [40]. The *B. burgdorferi* s.l. includes at least 20 species, with *B. burgdorferi* sensu stricto, *B. afzelii*, and *B. garinii* being the primary human pathogens [41,42]. These spirochetes demonstrate remarkable phenotypic plasticity, adapting their gene expression profiles to survive in different host environments (Figure 2). The characteristic erythema migrans rash occurs in 70–80% of cases, followed by potential disseminated infection involving joints, the heart, and the nervous system [43].

Rocky Mountain spotted fever and other spotted fever group rickettsioses are transmitted by various tick species and can cause severe, potentially fatal disease if untreated [44,45,46]. *Rickettsia rickettsii* invades endothelial cells, causing widespread vasculitis that can lead to capillary leak, thromocytopenia, and multi-organ failure [47]. The classic triad of fever, headache, and rash occurs in less than 60% of patients, making early diagnosis challenging. Other spotted fever group rickettsiae include *R. conorii* (Mediterranean spotted fever), *R. africae* (African tick bite fever), and *R. parkeri* (American boutonneuse fever) [48,49,50].

Anaplasmosis, caused by *Anaplasma phagocytophilum*, primarily affects neutrophils and can cause flu-like symptoms that can progress to severe multi-organ dysfunction [51,52]. The pathogen forms characteristic morulae within neutrophil cytoplasm and can persist in infected hosts for extended periods [53,54]. Ehrlichiosis encompasses several species, including *E. chaffeensis* (human monocytic ehrlichiosis) and *E. ewingii* (ehrlichiosis ewingii), each targeting specific white blood cell populations [55,56,57].

Tularemia, caused by *Francisella tularensis*, is a highly infectious bacterial disease transmitted by multiple tick species [58]. This Category A bioterrorism agent requires as few as 10 organisms that cause infection and can present in several clinical forms, including ulcerglandular, glandular, oculoglandular, oropharyngeal, pneumonic, and typhoidal. The bacteria can survive for weeks in water and soil, contributing to their persistence in natural environments.

### 3.2. Viral Pathogens

Tick-borne viral diseases pose increasing threats to human health, with several emerging or reemerging viruses causing severe disease (Table 1). These viruses belong to several families, including Flaviviridae, Bunyaviridae (now Peribunyaviridae and Phenuiviridae), and Reoviridae, each with distinct replication strategies and pathogenic mechanisms [59].

**Table 1:** Major tick-borne pathogens and their primary vectors.

Pathogen Type	Human Pathogen Species	Disease	Primary Tick Vector	Geographic Distribution	Ref.
Bacteria	*Borrelia burgdorferi* s.l.	Lyme disease	*Ixodes scapularis*, *I. ricinus*	North America, Europe, Asia	[39,42]
	*Rickettsia rickettsii*	Rocky Mountain spotted fever	*Dermacentor variabilis*, *D. andersoni*	Americas	[43,44]
	*Anaplasma phagocytophilum*	Human granulocytic anaplasmosis	*Ixodes scapularis*, *I. ricinus*	North America, Europe	[50,52]
	*Ehrlichia chaffeensis*	Human monocytic ehrlichiosis	*Amblyomma americanum*	Southeastern United States	[54,55]
	*Francisella tularensis*	Tularemia	*Dermacentor variabilis*, *Amblyomma americanum*	North America, Europe, Asia	[57]
	*Coxiella burnetii*	Q fever	Multiple tick species	Worldwide	[60,61]
Viruses	Tick-borne encephalitis virus	Tick-borne encephalitis	*Ixodes ricinus*, *I. persulcatus*	Europe, Asia	[59,62]
	Crimean-Congo hemorrhagic fever virus	Crimean-Congo hemorrhagic fever	*Hyalomma marginatum*	Africa, Asia, Europe	[63]
	Powassan virus	Powassan encephalitis	*Ixodes scapularis*, *I. cookei*	North America	[64]
	SFTS virus	Severe fever with thrombocytopenia syndrome	*Haemophysalis longicornis*	East Asia	[65]
Protozoa	*Babesia microti*	Human babesiosis	*Ixodes scapluris*	North America	[66,67]
	*Babesia divergens*	Human babesiosis	*Ixodes ricinus*	Europe	[67]
	*Theileria parva*	East Coast fever	*Rhipicephalus appendiculatus*	Eastern and Southern Africa	[12,68]

Tick-borne encephalitis virus (TBEV), endemic throughout Europe and Asia, causes severe neurological disease with significant mortality and long-term sequelae [62]. TBEV exists as three subtypes: European (TBEV-Eur), Siberian (TBEV-Sib), and Far Eastern (TBEV-FE), with the Far Eastern subtype associated with the highest case fatality rates (up to 35%) [63]. The virus causes a biphasic illness in about two-thirds of patients, with an initial flu-like phase followed by neurological involvement, including meningitis, encephalitis, and myelitis. Long-term neurological sequelae occur in 10–20% of patients, including cognitive impairment, motor deficits, and seizure disorders.

Crimean-Congo hemorrhagic fever virus (CCHFV), transmitted by *Hyalomma* ticks, causes severe hemorrhagic fever with high case fatality rates [64]. This Nairovirus can cause case fatality rates ranging from 10 to 40%, with death typically occurring within the first two weeks of illness. The virus causes capillary fragility, coagulopathy, and multi-organ failure. CCHFV has the widest geographic distribution of any tick-borne virus, extending across Africa, Asia, and southeastern Europe.

Powassan virus, transmitted primarily by *Ixodes* ticks in North America, can cause severe encephalitis with fatality rates up to 10% [65]. Unlike many tick-borne pathogens, Powassan virus can be transmitted within 15 min of tick attachment, making rapid tick removal less effective as a prevention strategy. Severe fever with thrombocytopenia syndrome virus (SFTSV), discovered in China in 2009, has emerged as a significant public health threat in East Asia [66]. This Phlebovirus causes a severe febrile illness with thrombocytopenia, leukopenia, and multi-organ dysfunction, with case fatality rates ranging from 6% to 30%.

Other important tick-borne viruses include Colorado tick fever virus (Coltivirus), Kyasanur Forest disease virus (Flavivirus), and Omsk hemorrhagic fever virus (Flavivirus). The emergence of new tick-borne viruses continues, with recent discoveries including Heartland virus and Bourbon virus in North America, highlighting the ongoing risk of novel viral zoonoses.

### 3.3. Protozoal Pathogens

Protozoal tick-borne diseases affect both humans and animals, with *Babesia* species representing the most important group of protozoa transmitted by ticks (Table 1). These apicomplexan parasites have complex life cycles involving both sexual reproduction in tick vectors and asexual reproduction in vertebrate hosts. The parasites invade red blood cells, causing hemolytic anemia and potentially life-threatening complications.

Human babesiosis, caused primarily by *Babesia microti* in North America and *Babesia divergens* in Europe, can cause severe disease, particularly in immunocompromised individuals [67]. *B. microti* typically causes milder disease than *B. divergens*, with many infections being asymptomatic or causing mild flu-like symptoms [68]. However, severe cases can develop hemolytic anemia, jaundice, hemoglobinuria, and renal failure. *B. divergens* infections are often more severe, particularly in splenectomized patients, with fulminant cases resembling falciparum malaria and carrying high mortality rates.

*Theileria* species, primarily veterinary pathogens, also have zoonotic potential and cause significant economic losses in livestock [69]. *T. parva* causes East Coast fever in cattle, characterized by lymphoproliferative disease with high mortality rates. *T. annulata* causes tropical theileriosis, while *T. orientalis* has emerged in new geographic areas, including Australia and New Zealand. Recent reports suggest some *Theileria* species may occasionally infect humans, particularly immunocompromised individuals.

Other protozoal tick-borne pathogens include *Cytauxzoon felis*, which causes cytauxzoonosis in domestic cats, and various *Hepatozoon* species that infect dogs and wild carnivores. *Babesia venatorum* (formerly *Babesia* sp. EU1) has emerged as a human pathogen in Europe, causing severe disease in some cases. The diversity of protozoal pathogens in tick populations likely exceeds current knowledge, as molecular surveys have revealed numerous undescribed species with unknown pathogenic potential.

## 4. Tick Microbiomes

### 4.1. Bacterial Components

The bacterial component of tick microbiomes represents the most diverse and well-characterized fraction, including both obligate endosymbionts and environmentally acquired bacteria (Table 2) [70,71,72,73,74,75,76]. These bacteria play crucial roles in tick physiology, immunity, and pathogen interactions, with implications for disease transmission dynamics [60].

*Coxiella*-like bacteria are widespread symbionts in many tick species, with some strains closely related to *Coxiella burnetii*, the causative agent of Q fever [61,77]. These intracellular bacteria reside primarily in tick ovaries and are maternally transmitted to offspring. They play essential roles in tick physiology, particularly in nutrition and reproduction, potentially supplementing essential amino acids and vitamins that are deficient in blood meals [78]. The phylogenetic relationship between *Coxiella*-like endosymbionts (CLEs) and pathogenic *C. burnetii* raises important questions about the evolution of pathogenicity and zoonotic potential.

*Candidatus* Midichloria mitochondrii represents a unique endosymbiont that colonizes the mitochondria of *Ixodes ricinus* ticks [79,80,81]. This bacterium is the only known organism to live consistently within mitochondria, representing a novel form of symbiosis. The functional significance of this association remains unclear, though it may influence tick metabolism and cellular physiology.

*Borrelia burgdorferi* s.l. is a complex of spirochete bacteria that causes Lyme disease, the most widely distributed tick-borne disease in the Northern Hemisphere. These spirochetes demonstrate remarkable adaptability, modifying their outer surface proteins to evade host immune responses and adapt to different host environments. The bacteria can persist in tick populations through transovarial and transstadial transmission, though efficiency varies among *Borrelia* species and tick populations.

*Rickettsia* species associated with ticks include both pathogenic and apparently non-pathogenic strains [82]. Some *Rickettsia* species appear to function as endosymbionts, enhancing tick fitness and reproduction, while others cause disease in vertebrate hosts. The distinction between pathogenic and endosymbiotic *Rickettsia* species is not always clear, and some strains may represent intermediate forms in the evolution from mutualism to parasitism.

Environmental bacteria acquired from hosts or habitats constitute a significant portion of tick microbiomes [83,84,85,86,87,88,89]. These include *Pseudomonas*, *Acinetobacter*, *Staphylococcus*, and *Bacillus* species that may be transiently associated with ticks [90,91]. While some may represent contamination, others could play roles in pathogen exclusion or enhancement through competitive or synergistic interactions [92].

### 4.2. Viral Components

The viral fraction of the tick microbiome, collectively termed the “tick virome,” represents a largely unexplored reservoir of potential zoonotic pathogens (Table 2). Recent advances in high-throughput sequencing have revealed that tick viromes are extraordinarily diverse, containing hundreds of novel viral species with unknown pathogenic potential.

Metagenomic studies have identified numerous novel viruses in tick populations, including representatives of families known to harbor important human pathogens [93]. These discoveries suggest that the current catalog of tick-borne viruses represents only a small fraction of the total viral diversity present in tick populations. Many novel viruses show distant relationships to known pathogens, making it difficult to predict their zoonotic potential solely from sequence similarity.

**Table 2:** Major microbial components of the tick microbiome.

Microbial Group	Representative Taxa	Tick Species	Function/Role	Pathogenic Potential	Ref.
Endosymbionts	*Candidatus* Midichloria mitochondrii	*Ixodes ricinus*	Mitochondrial symbiont, unknown function	Unknown	[80,81]
	*Coxiella*-like endosymbionts	Multiple tick species	Nutrition, reproduction	Low to moderate	[61,77]
	*Rickettsia* endosymbionts	Various tick species	Fitness enhancement, reproduction	Variable	[82]
	*Francisella*-like endosymbionts	*Dermacentor*, *Amblyomma*	Nutritional supplementation	Unknown	[84]
	*Wolbachia* spp.	Various arthropod parasitoids	Reproductive manipulation	None	[94]
	*Arsenophonus* spp.	Multiple tick species	Unknown symbiotic role	Unknown	[82]
Environmental Bacteria	*Pseudomonas* spp.	Multiple tick species	Environmental acquisition	Low	[61,85,89]
	*Sphingomonas* spp.	Various tick species	Environmental containment	None	[61,85,89]
	*Acinetobacter* spp.	Multiple tick species	Environmental acquisition	Low	[61,85,89]
	*Staphylococcus* spp.	Various species	Commensal bacteria	Variable	[61,85,89]
Viral Components	Novel RNA viruses	Multiple tick species	Unknown	Unknown	[93,95,96]
	Tick-specific bacteriophages	Various tick species	Bacterial population regulation	None	
	Arthropod-specific viruses	Multiple tick species	Potential immune modulation	Low	[70,74,90]

RNA viruses, including flaviviruses, bunyaviruses, and rhabdoviruses, constitute important components of the tick virome [95,96]. Flaviviruses are among the most important tick-borne pathogens, including the tick-borne encephalitis virus and the Powassan virus. Novel flavivirus-like sequences have been detected in multiple tick species, suggesting the existence of undiscovered pathogenic flaviviruses. Bunyaviruses (now classified into multiple families) include Crimean-Congo hemorrhagic fever virus and SFTS virus, both of which cause several human diseases.

Tick-specific viruses that appear to infect only arthropods constitute another important component of tick viromes [97]. These include insect-specific flaviviruses, rhabdoviruses, and members of newly discovered viral families. While these viruses may not directly infect vertebrates, they could influence tick physiology, immunity, and pathogen transmission competence. Some arthropod-specific viruses may serve as evolutionary precursors to vertebrate-pathogenic viruses.

Bacteriophages targeting tick-associated bacteria represent another viral component that could influence microbiome composition and pathogen transmission. These phages may regulate bacterial populations within ticks, potentially affecting the abundance of pathogenic or beneficial bacteria. The ecological roles of tick-associated bacteriophages remain largely unexplored but could represent novel targets for microbiome manipulation strategies.

### 4.3. Protozoa Components

Protozoal diversity within tick microbiomes extends beyond known pathogenic species to include numerous undescribed organisms of unknown significance. The application of molecular techniques to tick-borne pathogen surveillance has revealed that protozoal diversity in ticks far exceeds previous estimates based on morphological and classical molecular approaches.

Environmental surveys have revealed extensive diversity of *Babesia*-like and *Theileria*-like organisms in tick populations, many of which are associated with wildlife hosts. Phylogenetic analyses suggest the existence of numerous cryptic species within established genera, as well as potentially novel genera of tick-borne protozoa. Many of these organisms show host-specific associations with particular wildlife species, suggesting co-evolutionary relationships that may influence their zoonotic potential.

The potential for these organisms to infect humans or livestock is largely unknown, representing important knowledge gaps in zoonotic disease risk assessment. Experimental infections and serological surveys are needed to determine the host range and pathogenic potential of newly discovered protozoal species. Some organisms that appear benign in their natural wildlife hosts may cause severe disease when transmitted to naïve hosts, as demonstrated by the emergence of *Babesia venatorum* as a human pathogen.

*Hepatozoon* species represent another group of tick-transmitted protozoa that is gaining increasing recognition. While primarily known as pathogens of dogs and sild carnivores, some *Hepatozoon* species may have broader host ranges than previously recognized. The life cycle of many *Hepatozoon* species remains incompletely understood, complicating efforts to assess their public health significance.

Undescribed protozoal groups detected through environmental DNA surveys highlight that major gaps remain in our understanding of tick-borne protozoal diversity. Some sequences show only distant relationships to known protozoa, suggesting the existence of novel taxonomic groups. The ecological roles and pathogenic potential of these organisms represent important frontiers for future research.

## 5. Impact of Tick Microbiome on Transmissibility

The composition and dynamics of tick microbiomes significantly influence pathogen acquisition, maintenance, and transmission through complex ecological and physiological mechanisms (Table 3). These interactions represent a critical yet underappreciated factor in tick-borne disease epidemiology, with potential implications for disease prediction, prevention, and control [98,99].

Symbiotic bacteria can enhance or inhibit pathogen establishment through multiple mechanisms, including resource competition, production of antimicrobial compounds, or modulation of the tick immune response [102,103]. Resource competition occurs when microorganisms compete for limited nutrients, cellular attachment sites, or metabolic resources within the tick [100]. For example, *Rickettsia* endosymbionts and pathogenic *Rickettsia* species may compete for similar intracellular niches, potentially reducing pathogen loads through competitive exclusion.

Antimicrobial compound production by microbiome members can directly inhibit pathogen growth and survival. Some *Pseudomonas* species isolated from ticks produce bacteriocins and other antimicrobial compounds that can suppress the growth of *B. burgdorferi* s.l. in laboratory conditions. However, the relevance of these interactions under natural conditions remains to be established.

Immune modulation represents another important mechanism by which microbiomes influence pathogen transmission [104]. Diverse microbial communities can prime tick immune responses, making them more resistant to subsequent pathogen invasion [101]. Conversely, some microorganisms may suppress immune responses, creating more permissive environments for pathogen establishment. The immunomodulatory effects of specific microbiome members are beginning to be characterized through transcriptomic and functional studies [105].

Coinfections with multiple pathogens can result in synergistic or antagonistic interactions that affect transmission efficiency [106,107]. Synergistic interactions may occur when one pathogen facilitates the establishment or transmission of another, potentially through immune suppression or metabolic modification of the tick environment [108]. Antagonistic interactions may result from direct competition or indirect effects mediated through host immune responses.

The temporal dynamics of microbiome-pathogen interactions add another layer of complexity. Microbiome composition changes throughout tick development and feeding, potentially altering susceptibility to pathogen infection at different life stages. Blood meal acquisition introduces transient microbial communities that may interact with established microbiomes and influence pathogen transmission dynamics.

Environmental factors, including temperature, humidity, and host species, can influence both microbiome composition and pathogen–microbiome interactions. Climate change may alter these relationships in unpredictable ways, potentially affecting tick vector competence and disease transmission patterns. Understanding these complex interactions will be crucial for predicting future changes in tick-borne disease risk.

## 6. Surveillance and Vaccine Development

### 6.1. Field Screening Approaches

Rapid field identification of tick-borne pathogens is essential for the timely diagnosis and treatment of tick-borne diseases (Table 4). Current laboratory-based diagnostic approaches often take days to weeks to yield results, delaying critical treatment decisions and public health responses. The development of field-deployable diagnostic technologies is a priority for reducing the impact of tick-borne diseases.

Field-deployable diagnostic devices that utilize isothermal amplification techniques, such as loop-mediated isothermal amplification (LAMP), show promise for pathogen detection [109]. LAMP reactions can be performed at constant temperatures (60–65 °C) using simple heating devices, eliminating the need for thermal cycling equipment [110]. Visual detection methods using colorimetric or fluorescent indicators allow results to be interpreted without specialized equipment, making LAMP particularly suitable for resource-limited settings.

Lateral flow immunoassays and biosensor technologies offer potential for rapid screening of tick specimens or clinical samples [111]. These technologies can provide results within 15–30 min and require minimal training to operate. Recent advances in signal amplification and multiplex detection capabilities have improved sensitivity and specificity, making these approaches increasingly viable for field use.

Portable PCR devices and real-time detection systems are becoming increasingly sophisticated while maintaining field portability [112,113]. These systems can provide quantitative results with sensitivity comparable to that of laboratory-based methods. Integration with smartphone applications and cloud-based data management systems enables real-time data sharing and epidemiological analysis.

Environmental DNA (eDNA) approaches offer potential for habitat-based pathogen surveillance without requiring tick collection [122,123]. Water, soil, and vegetation samples can be analyzed for pathogen DNA, providing information about pathogen presence in specific habitats. This approach could be particularly valuable for monitoring remote or inaccessible areas and for large-scale surveillance programs.

### 6.2. Vaccine Development

Anti-tick vaccines represent a promising approach for reducing tick populations and disrupting pathogen transmission cycles [114,115]. This approach offers several advantages over traditional acaricide-based control methods, including reduced environmental impact, absence of resistance development, and potential for long-lasting protection. Anti-tick vaccines work by inducing antibodies that interfere with tick feeding, digestion, or reproduction when ticks feed on vaccinated hosts.

Vaccines targeting tick feeding and reproduction can reduce tick survival and fertility on vaccinated hosts [117]. Feeding-blocking vaccines target proteins involved in blood digestion, attachment, or feeding behavior, while reproduction-targeting vaccines focus on proteins essential for egg development or fertility. The most successful anti-tick vaccine to date, TickGARD/Gavac, targeting *Rhipicephalus microplus*, demonstrated significant reductions in tick populations and pathogen transmission in cattle.

Identification of conserved tick antigens essential for feeding or reproduction provides targets for broad-spectrum tick vaccines [116]. Comparative genomic and proteomic approaches have identified numerous candidate antigens conserved across multiple tick species. These include enzymes involved in blood digestion, structural proteins of the feeding apparatus, and hormones regulating development and reproduction.

Multi-pathogen vaccines targeting several tick-borne diseases simultaneously represent an attractive approach for comprehensive protection [118,119]. Such vaccines could combine antigens from multiple pathogens or use platform technologies that provide broad-spectrum immunity. Recent advances in vaccine adjuvants and delivery systems have improved the feasibility of multi-component vaccines [120].

Microbiome-based vaccine approaches represent a novel frontier in the prevention of tick-borne diseases [121]. These could include vaccines targeting key microbiome members that facilitate pathogen transmission or vaccines designed to promote beneficial microbiome communities that inhibit pathogen establishment. While still largely conceptual, this approach could provide highly targeted interventions with minimal ecological disruption.

## 7. Conclusions

Ticks are globally important vectors of zoonotic disease transmission, and their importance continues to increase due to climate change, landscape changes, and expanding human–wildlife interfaces. The convergence of environmental, ecological, and social factors has created conditions favoring tick population expansion and increased human exposure risk. Understanding these complex interactions is crucial for predicting and managing future tick-borne disease threats.

The diversity of pathogens transmitted by ticks exceeds that of most other arthropod vectors, including bacteria, viruses, protozoa, and other microorganisms with varying degrees of pathogenicity. This extraordinary pathogen diversity reflects the unique ecological niche occupied by ticks as long-lived, multi-host parasites that can maintain microbial communities across extended periods and diverse environmental conditions.

The complex microbial communities associated with ticks include numerous undescribed organisms with unknown zoonotic potential. The tick microbiome represents a vast reservoir of genetic diversity shaped by millions of years of coevolution with diverse host species and environmental conditions. This diversity encompasses not only potential pathogens but also beneficial symbionts and environmentally acquired microorganisms that collectively influence tick biology and pathogen transmission.

Recent advances in molecular biology have revealed that tick microbiomes are far more diverse than previously recognized, suggesting that many potential zoonotic pathogens remain undiscovered. High-throughput sequencing technologies have revolutionized our understanding of microbial diversity, revealing complex communities that were invisible to traditional culture-based approaches. However, the functional significance of most microbiome components remains unknown, representing a major knowledge gap in our understanding of tick-borne disease ecology.

The interactions between tick microbiomes and pathogen transmission represent a critical but underexplored aspect of tick-borne disease ecology. These interactions can dramatically influence pathogen acquisition, maintenance, and transmission efficiency, with implications for disease risk assessment and control strategies. Future research must integrate microbiome ecology with traditional epidemiological approaches to develop a more comprehensive understanding of tick-borne disease dynamics.

Climate change and environmental degradation are likely to alter the tick-pathogen microbiome interactions in unpredictable ways. Rising temperatures may favor certain microbiome members while suppressing others, potentially altering pathogen transmission dynamics. Geographic range expansions of tick species may introduce novel pathogen–microbiome combinations with unknown consequences for disease emergence.

Priority areas for future research include the development of rapid diagnostic tools for tick-borne pathogens, comprehensive characterization of tick microbiome diversity, and assessment of zoonotic potential among tick-associated microorganisms. Functional studies are needed to determine the roles of specific microbiome members in pathogen transmission and tick biology. Long-term monitoring programs should integrate microbiome analysis with traditional surveillance to detect emerging threats.

**Figure 3:**
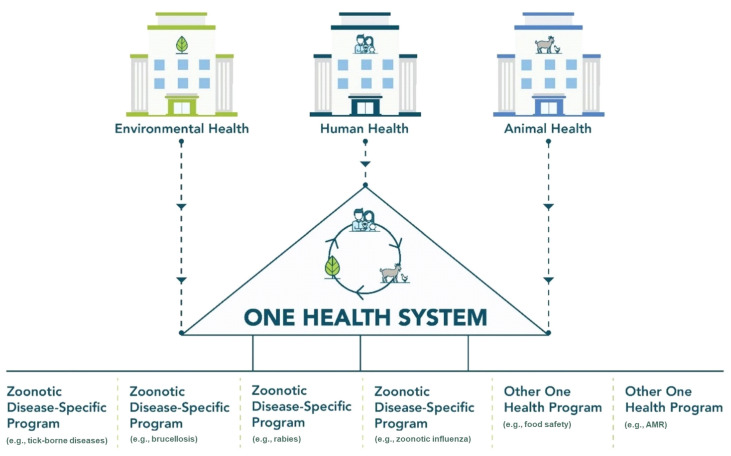
One Health approach for zoonotic disease control. Diagram showing a comprehensive One Health framework for zoonotic disease control. Emphasizes the interconnectedness of human, animal, and environmental health and the need for integrated surveillance and response strategies. Source: Nature Scientific Reports (https://doi.org/10.1038/s41598-022-12619-1) [124].

Implementation of One Health surveillance approaches integrating human, animal, and environmental monitoring will be essential for detecting emerging tick-borne diseases (Figure 3). Such approaches must incorporate microbiome analysis and environmental monitoring to provide early warning of changing disease risks. Cross-sector collaboration and data sharing will be crucial for the effective implementation of integrated surveillance systems.

The development of novel prevention strategies, including tick vaccines and microbiome-based interventions, offers promise for reducing future tick-borne disease burden. These approaches may offer more sustainable, environmentally friendly alternatives to traditional chemical control methods. However, successful implementation will require a better understanding of tick–microbiome–pathogen interactions and their ecological consequences.

The global burden of tick-borne diseases will likely continue to increase in the coming decades due to environmental and social changes. Proactive research investments in tick microbiome ecology, pathogen discovery, and novel control strategies are essential for mitigating future disease impacts. International cooperation and standardized approaches will be crucial for addressing the global nature of tick-borne disease threats.

Ultimately, effective management of tick-borne disease risks requires integrating diverse scientific disciplines, from microbiology and ecology to public health and the social sciences. The complexity of tick-borne disease systems demands comprehensive, interdisciplinary approaches that recognize the interconnected nature of environmental, animal, and human health. Only through such integrated efforts can we reduce the growing burden of tick-borne diseases on global health and well-being.

## Figures and Tables

**Table 3:** Microbiome effects on tick-borne pathogen transmission.

Microbiome Component	Target Pathogen	Effect on Transmission	Mechanism	Evidence Level	Ref.
*Rickettsia buchneri*	*Borrelia burgdorferi* s.l.	Enhancement	Immune suppression, resource provision	Experimental	[100]
*Candidatus* Midichloria	Multiple pathogens	Variable	Metabolic interference	Observational	[80,81]
Native bacterial community	*Anaplasma phagocytophilum*	Inhibition	Resource competition, antagonism	Laboratory	[100]
*Pseudomonas* spp.	*Borrelia burgdorferi* s.l.	Inhibition	Antimicrobial compound production	In vitro	[101]
Diverse microbiome	Tick-borne encephalitis virus	Inhibition	Immune activation, resource competition	Field studies	[97]
*Coxiella*-like endosymbionts	*Rickettsia* spp.	Competition	Niche overlap, resource limitation	Correlational	[61,82]
Antibiotic-depleted microbiome	Multiple pathogens	Enhancement	Reduced microbial competition	Experimental	[101]
*Enterobacter* spp.	*Babesia microti*	Enhancement	Metabolic support, immune modulation	Preliminary	[101]
Novel RNA viruses	Bacterial pathogens	Variable	Immune interference, cellular stress	Theoretical	[95]
Blood meal microbiome	Multiple pathogens	Modulation	Temporary microbial influx	Observational	[78]

**Table 4:** Emerging technologies for tick-borne pathogen detection and control.

Technology Category	Specific Technology	Application	Advantages	Current Status	Ref.
Diagnostic Technologies	LAMP (Loop-mediated isothermal amplification)	Field-deployable pathogen detection	Rapid, isothermal, visual results	Research/Development	[109,110]
	Lateral flow immunoassays	Point-of-care diagnosis	Simple, rapid, no equipment needed	Commercial/Development	[111]
	Portable PCR devices	Field-based molecular detection	High sensitivity and specificity	Commercial	[112,113]
	Biosensor arrays	Multiplex pathogen screening	Simultaneous multiple target detection	Research	
Vaccine Development	Anti-tick vaccines	Tick population control	Targets multiple pathogens	Research/Trials	[114,115,116]
	Multi-pathogen vaccines	Broad-spectrum protection	Single vaccine, multiple diseases	Development	[117,118,119,120]
	Microbiome-based vaccines	Pathogen transmission blocking	Target transmission mechanisms	Conceptual	[121]
Surveillance Technologies	Environmental DNA (eDNA)	Habitat-based pathogen monitoring	Non-invasive, large-scale screening	Research	[122,123]
	Remote sensing integration	Risk prediction modeling	Large-scale risk assessment	Development	
	AI-powered image analysis	Automated tick identification	Rapid species identification	Development	
Control Strategies	Microbiome manipulation	Pathogen transmission reduction	Targeted, ecological approach	Research	[99,101]
	Sterile insect technique adaptation	Tick population suppression	Species-specific control	Conceptual	

## Data Availability

No new data were created or analyzed in this study. Data sharing is not applicable to this article.

## Abbreviations

The following abbreviations are used in this manuscript:

PHIL	Public Health Image Library
TBEV	Tick-borne encephalitis
CCHFV	Crimean-Congo hemorrhagic fever virus
SFTSV	Severe fever with thrombocytopenia syndrome virus
CLEs	*Coxiella*-like endosymbionts
LAMP	Loop-mediated isothermal amplification
eDNA	Environmental DNA
